# Effects of the G-quadruplex-binding drugs quarfloxin and CX-5461 on the malaria parasite *Plasmodium falciparum*

**DOI:** 10.1016/j.ijpddr.2023.11.007

**Published:** 2023-11-24

**Authors:** Holly M. Craven, Guilherme Nettesheim, Pietro Cicuta, Andrew M. Blagborough, Catherine J. Merrick

**Affiliations:** a Department of Pathology, University of Cambridge, Tennis Court Road, Cambridge, CB2 1QP, UK; b Department of Physics, Cavendish Laboratory University of Cambridge, J.J. Thomson Avenue, Cambridge, CB3 0HE, UK

**Keywords:** Malaria, *Plasmodium*, G-quadruplex, quarfloxin, CX-5461, repositioning

## Abstract

*Plasmodium falciparum* is the deadliest causative agent of human malaria. This parasite has historically developed resistance to most drugs, including the current frontline treatments, so new therapeutic targets are needed. Our previous work on guanine quadruplexes (G4s) in the parasite's DNA and RNA has highlighted their influence on parasite biology, and revealed G4 stabilising compounds as promising candidates for repositioning. In particular, quarfloxin, a former anticancer agent, kills blood-stage parasites at all developmental stages, with fast rates of kill and nanomolar potency. Here we explored the molecular mechanism of quarfloxin and its related derivative CX-5461. *In vitro,* both compounds bound to *P. falciparum*-encoded G4 sequences. *In cellulo*, quarfloxin was more potent than CX-5461, and could prevent establishment of blood-stage malaria *in vivo* in a murine model. CX-5461 showed clear DNA damaging activity, as reported in human cells, while quarfloxin caused weaker signatures of DNA damage. Both compounds caused transcriptional dysregulation in the parasite, but the affected genes were largely different, again suggesting different modes of action. Therefore, CX-5461 may act primarily as a DNA damaging agent in both *Plasmodium* parasites and mammalian cells, whereas the complete antimalarial mode of action of quarfloxin may be parasite-specific and remains somewhat elusive.

## Highlights

• The G4 stabilising compound quarfloxin is an effective antimalarial, both *in vitro* on human malaria parasites and *in vivo* on murine malaria parasites.
• The related compound CX-5461, currently in trials against cancer, acts primarily to cause DNA damage in *Plasmodium* parasites, as it does in human cells.
• The antimalarial mechanism of quarfloxin is not identical to that of CX-5461, being more toxic but less DNA damaging: it is probably *Plasmodium*-specific and multi-factorial.

## Graphical abstract

**Figure:**
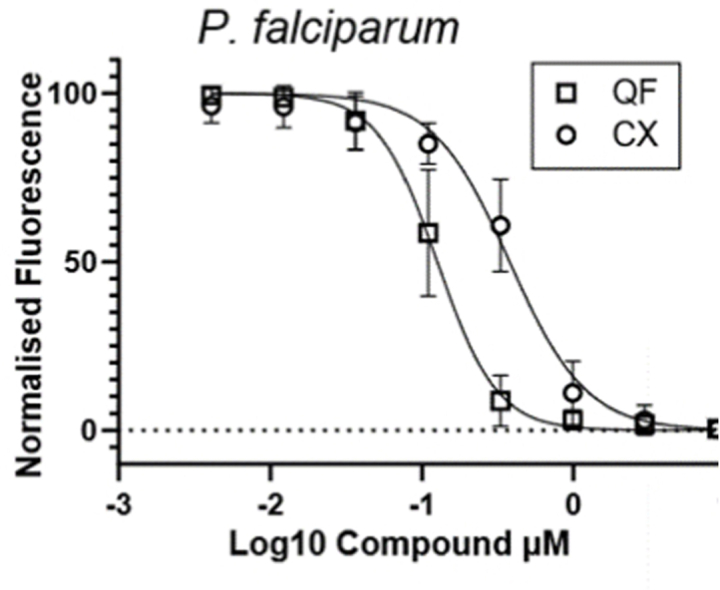

## 1 Introduction

Malaria is a disease caused by infection with parasitic apicomplexan *Plasmodium* species, transmitted through bites of female *Anopheles* mosquitoes. The disease is prevalent throughout tropical and sub-tropical regions of Africa, Asia, South America and Oceania and causes significant morbidity and over half a million deaths annually (World Health Organization, 2022). Of the six species known to cause human malaria, *Plasmodium falciparum* causes the most deaths. *P. falciparum* is endemic throughout sub–Saharan Africa and much of Asia, and the disease is a major barrier to socio-economic development (Gallup and Sachs, 2001). Despite being managed in many parts of the world through a rigorous programme of insecticide spraying, insecticide-treated bed net distribution and antimalarial treatment, resistance to both insecticides and drugs commonly arises (Balikagala et al., 2021; Dondorp et al., 2009; Uwimana et al., 2020; Yeung et al., 2004; Zhu et al., 2022) and can spread rapidly (Hamilton et al., 2019; Sonhafouo-Chiana et al., 2022; Weedall et al., 2020). Although current programmes of artemisinin-based combination therapy (ACT) have greatly reduced the clinical burden of malaria, (WHO, 2022) new drug targets are needed, especially as ACT failure is prevalent in S.E. Asia and parasite resistance has now been reported in Africa (Balikagala et al., 2021; Fairhurst and Dondorp, 2016; Silva-Pinto et al., 2021).

Here, we focus on the guanine quadruplex (G4) as a potential antimalarial target. Being a nucleic acid structure rather than a protein, the G4 would represent an entirely novel drug target in the parasite. *P. falciparum* has a highly A/T-biased genome (∼81% A/T) (Gardner et al., 2002) with very few sequences that have the potential to form G4s (‘PQSs’); nevertheless, we and others have shown that these G4s do exist and that they play roles in *Plasmodium* biology at both the DNA (Gage and Merrick, 2020; Gazanion et al., 2020; Harris et al., 2018; Smargiasso et al., 2009) and RNA (Dumetz et al., 2021; Edwards-Smallbone et al., 2022) levels. The existence of G4s in malaria parasites raises the possibility of repositioning G4-binding compounds – which are often developed primarily as anticancer agents – as antimalarial agents. Repositioning can be a valuable strategy for tropical disease treatment, due to limited funds for novel compound development, and it has previously achieved success; notably in the case of doxycycline, an antibiotic, used commonly as a prophylaxis for travellers to malaria endemic regions (Berenstein et al., 2016; Charlton et al., 2018; Joachim Haupt and Schroeder, 2011). While no G4 targeting compounds have been clinically approved as yet, there is growing interest in the field due to their associations with cancer. In this work, we focussed on using these G4-targeting compounds as potential ways to elucidate mechanistic pathways and interactions within the parasite.

We previously showed that a G4-binding agent called quarfloxin, which previously reached phase IIa clinical trials for cancer (Drygin et al., 2009, 2008; Lim et al., 2009; K. K. Papadopoulos et al., 2007; K. P. K. P. Papadopoulos et al., 2007), is potent (EC_50_ 110–130 nM) and fast-acting against *P. falciparum* parasites cultured *in vitro* (Harris et al., 2018). However, the compound did not act mechanistically via the route characterised in human cells, and subsequently also in trypanosomes (Brooks and Hurley, 2010; Drygin et al., 2008, 2009; Kerry et al., 2017), i.e. disrupting rRNA production by RNA polymerase I. This was perhaps unsurprising, since rRNA genes in *Plasmodium* do not occur in the characteristic tandem arrays found in many eukaryotes (Mancio-Silva et al., 2010). We also established that although quarfloxin is a fluoroquinolone, structurally related to the 4-aminoquinolines, it showed no activity in inhibiting haem crystallisation – the main mode of action for drugs like chloroquine. Furthermore, it was highly potent against rings, causing cell death and failure to progress to trophozoite stages (Harris et al., 2018), which is also inconsistent with a chloroquine-like mode of action. Importantly, all this suggested that quarfloxin may have a parasite-specific mode of toxicity, which could lead to new mechanistic insights into both drug-parasite interactions and potential pathways regulated by G4s that might differ between host and parasite. Here, we set out to characterise the anti-parasitic mode of action for both quarfloxin and a related compound CX-5461 (brand-named Pidnarulex), which remains under investigation as an anticancer chemotherapy (Bruno et al., 2020; Yan et al., 2021) to see if mechanisms of action might differ between these related compounds.

We report that quarfloxin and CX-5461 both interact with quadruplex-forming sequences of parasite DNA, and that they both cause some signatures of DNA damage. However, this may not be the sole mode of action, particularly for quarfloxin. CX-5461 is a more effective DNA damaging agent than quarfloxin, yet quarfloxin is more toxic to parasites than CX-5461. To gain more insight into this difference, we also explored the acute effects of both compounds on the trophozoite transcriptome and found distinct signatures of transcriptional disruption, with several PQS-containing genes affected. CX-5461 was heavily associated with disrupting replication and erythrocyte-invasion pathways, while quarfloxin disrupted fewer genes, including those in variantly-expressed sub telomeric gene families and those involved in the heatshock stress response. Overall, we find differing mechanisms of action for these compounds, although both function as DNA damaging agents in *Plasmodium*.

## 2 Materials and methods

### 2.1 Compound preparation and storage

Compounds were solubilised and stored in the dark at −20 °C: quarfloxin at 1 mM in ultrapure water, CX-5461 at 1.5 mM in DMSO.

### 2.2 Parasite culture and lines

*P. falciparum* 3D7 parasites were cultured *in vitro* in customised RPMI 1640 medium (2.3 g/L sodium bicarbonate, 4 g/L dextrose, 5.957 g/L HEPES, 0.05 g/L hypoxanthine, 5 g/L Albumax II, Invitrogen) supplemented with 200 mM L-glutamine, 25 μg gentamicin and 2.5% human serum. Parasites were maintained at 4% haematocrit in human O^+^ erythrocytes at 37 °C under specific gas mixture (3% O_2_, 5% CO_2_, 92% N_2_). *P. knowlesi* were maintained similarly, supplemented with 10% horse serum (Gibco 26050088) instead of human serum and 22.2 mM glucose, and maintained at 2% haematocrit. The Rad54 WT and Mut lines (Dumetz et al., 2021), *Pf*BLM and *Pf*WRN knockout/knockdown lines (Claessens et al., 2018) and the strain expressing thymidine kinase (Merrick, 2015) are previously described. For long term quarfloxin tolerance studies, 3D7 parasites were constantly cultured in the presence of sublethal doses of quarfloxin as determined by previous EC_50_ calculations (Harris et al., 2018). Tolerance was checked at intervals of several months by new Malaria SYBR green I based fluorescence (MSF) assays. Cultures that displayed promising quarfloxin tolerance were subject to clonal dilutions and then repeated MSF assays.

### 2.3 *P. berghei in vivo* experiments

All procedures were performed in accordance with the UK Animals (Scientific Procedures) Act (PP8697814) and approved by the University of Cambridge AWERB. The Office of Laboratory Animal Welfare Assurance for the University of Cambridge covers all Public Health Service supported activities involving live vertebrates in the US (no. A5634-01). This study was carried out in compliance with the ARRIVE guidelines (https://arriveguidelines.org/). Naïve CD1 mice (Charles River) (n = 5 per treatment) were infected by IP injection of 1 × 10^5^ *P. berghei* ANKA 2.34 parasites obtained from the blood of a donor mouse. Quarfloxin was dissolved in dH_2_O, and chloroquine (positive control) was dissolved in 7% Tween 80/3% ethanol in dH_2_O and dosed either intraperitoneally or intravenously to infected mice to result in a 25 mg/kg exposure for both compounds. Five additional infected mice received a vehicle-only dose as a negative control. Infected mice then received identical doses of compounds/vehicle 0, 24, 48 and 72 h after infection and, commencing at 96 h post-infection, were examined daily by withdrawal of blood from a peripheral tail vein, followed by Giemsa smear and microscopic examination to monitor parasitaemia. Mice reached their humane endpoint immediately upon showing a positive smear for malaria parasites and demonstrating outward pathological indications of malarial infection (e.g. piloerection, hunched posture, depressed desperation and ataxia). Mice were considered cured if this was not reached by day 30 post-infection. Mice dosed IP with 25 mg/kg quarfloxin demonstrated no sign of blood stage parasitaemia 96 h post-infection, but exhibited detrimental pathology and were humanely euthanised at this point to prevent breaching humane endpoint criteria.

### 2.4 Parasite synchronisation

Synchronised parasites were obtained by synchronisation 40 h apart with 5% sorbitol (w/v) to collect ring stages (Lambros and Vanderberg, 1979), or by 65% v/v percoll in PBS gradient (GE Healthcare) to collect schizonts (Rivadeneira et al., 1983). Tightly synchronised parasites (2 h window) were created by incubating late schizont stages with 1.5 μM protein kinase G inhibitor 4-[7-[(dimethylamino)methyl]-2-(4-fluorphenyl)imidazo [1,2-α]pyridine-3-yl]pyrimidin-2-amine (working name ‘compound 2’) (Baker et al., 2017) for 2 h before washing off with incomplete (serum-free) media. Reinvasion was allowed for 2 h in 25% haematocrit red blood cells as described previously (Totañes et al., 2023).

### 2.5 MSF assay

Malaria SYBR green I based fluorescence (MSF) assay was performed as previously described (Smilkstein et al., 2004). 96 well black walled plates (Greiner) were predosed with a 3× serial dilution of relevant compound, followed by addition of trophozoite cultures, in triplicate, to a final 0.5% parasitaemia, 2% haematocrit. Each plate contained triplicate wells without compound as negative control, 1 μM chloroquine as positive control, as well as triplicate wells of 2% uninfected haematocrit suspension for background correction. Outer wells contained culture media to prevent edge evaporation. Plates were incubated at 37 °C for 48 h. Following incubation, samples were mixed 1:1 with 2× MSF lysis buffer (20 mM Tris, pH 7.5, 5.5 mM EDTA, 0.008% saponin, 0.8% Triton-x-100) containing 0.2 μL/mL SYBR green I (Sigma) and transferred to a black walled plate. Following 1 h incubation at room temperature in the dark, fluorescence was detected using a POLAR Star OMEGA (BMG) microplate reader (ex 490 nm; em 510–570 nm). Normalised dose response curves and EC_50_ values with 95% confidence intervals (CI) were generated from blank-corrected means from three biological repeats using GraphPad Prism (v8) software (GraphPad Software Inc., San Diego, CA).

### 2.6 Detection of quarfloxin in live-cell microscopy

Live (unfixed) *P. falciparum* infected erythrocytes were incubated with 1 μM quarfloxin and either 50 nM DRAQ5 (Abcam) or 100 nM Lysotracker DeepRed (ThermoFisher) or MitoTracker Deep Red FM (ThermoFisher) for 30 min in the dark at 37*°*C, washed in PBS, diluted 100-fold in PBS and mounted onto SecureSeal hybridisation chambers (Grace Biol-Labs). Cells were imaged using a Nikon Eclipse Ti-E inverted microscope, temperature-controlled at 37 °C, with a Nikon Plan Apo VC 60×/1.40 oil objective and a CMOS camera (model GS3-U3-23S6M-C, Point Grey Research/FLIR Integrated Imaging Solutions (Machine Vision), Ri Inc., Canada). Quarfloxin was excited via Luxeon UV LHUV-0380-0200, peak at 380 nm, using the following Semrock filters: FF01-390/40 excitation filter, FF520-Di02 dichroic, FF01-542/27 emission filter. The companion dyes, DRAQ5, Lysotracker DeepRed and MitoTracker Deep Red, were excited via Luxeon Z LXZ1-PD01 at 627 nm using the following Semrock filters: FF01-534/635–25 excitation filter, FF560/659-Di01 dichroic, FF01-577/690–25 emission filter. Colocalisation analysis was performed in ImageJ (Schindelin et al., 2012).

### 2.7 TUNEL assay

Cultured parasites were incubated with 2× EC_50_ compound for 4 h and thin blood smears were created on glass slides. Samples were left to air dry, and then processed using *in situ* Cell Death Detection Kit TMR red (Roche) according to kit conditions and counterstained with 5 μg/mL DAPI prior to mounting with Prolong Diamond antifade mountant (Invitrogen). Samples were imaged using a Nikon Microphot SA microscope using blue (DAPI) and red (TMR-red) filters at 60× magnification. Nuclei were counted and percentages of TUNEL stained nuclei were calculated. 80–100 nuclei were counted for each treatment and three biological repeats were performed.

### 2.8 Western blot

Synchronous cultures at either ring, trophozoite or schizont stages were exposed to 2× EC_50_ compound for 2 h and parasites were saponin-released, lysed in RIPA buffer (150 mM NaCl, 50 mM Tris HCl, 1% v/v NP-40, 1 mM EDTA, 0.5% w/v sodium deoxycholate, 0.1% v/v SDS, 0.01% w/v sodium azide, pH 7.4), mixed with 4× LDS buffer (BioRad) and 5% v/v 2-mercaptoethanol and boiled for 5 min at 95 °C. Samples were electrophoresed on a 4–12% denaturing polyacrylamide gel (BioRad) in TGS buffer at 10 V/cm. The gel was transferred to a nitrocellulose membrane (Amersham) for 60 min at 60 V in chilled Towbin buffer. Following 1 h blocking with 3% BSA (w/v) 0.1% TBS-Tween 20, the blot was probed overnight with either 1:1000 anti-phospho-histone-H2A.X Ser 139 (#9718, CellSignalling) or anti H4 (ab10158, Abcam), rinsed, then probed with 1:2000 goat anti rabbit IgG-HRP (Abcam). Blots were developed using Clarity Western ECL substrate (Bio-Rad) and imaged using a GelDoc imager (Azure Biosystems). Following background correction and loading normalisation, densitometry analysis was performed in ImageJ (Schindelin et al., 2012) and the percentage increase in H2A-P signal was calculated relative to untreated samples.

### 2.9 ELISA

Early-trophozoite stage cultures were pulsed with high (10× EC_50_) and low (2× EC_50_) compound doses for 1 h and chased with 100 μM BrdU (Sigma Aldrich, B5002) for 1 h. Parasites were saponin released from erythrocytes, washed and resuspended in PBS and then aliquoted in triplicate into clear flat bottom 96 well plates and left to dry at 37 °C. A black walled plate was aliquoted with the same volume of parasites, mixed with an equal volume of 2× MSF lysis buffer containing 0.2 μL/mL SYBR green I and left to lyse for 1 h in the dark prior to reading on a platereader as per MSF assay protocol. This DNA measurement was then used to normalise the ELISA results for genomes loaded per well. After drying, parasites for ELISA were fixed for 15 min in 4% PFA and then 1:1 methanol: acetone for 2 min. Wells were rinsed three times with PBS for 5 min before blocking for 1 h in 1% BSA (w/v) PBS. BrdU was detected with 1:500 mouse anti BrdU (MoBU sc-51514, Santa Cruz) and 1U nuclease in 1% BSA. The plate was sealed and incubated for 1 h at 37 °C. Following 3 × 5 min rinses with PBS, wells were incubated with 1:5000 goat anti-mouse HRP (Dako) and incubated for 1 h in the dark. Following 3 × 5 min PBS rinses, TMB peroxidase (BioRad) was added and the plate was imaged on a POLAR Star Omega at 620 nm following 5 and 10 min development.

### 2.10 Thioflavin-T quadruplex-binding assays

ThT G4 binding assays were performed as described previously (Edwards-Smallbone et al., 2022). To allow for G4 folding, 40 μM synthetic DNA oligonucleotides (Sigma Aldrich) with 100 μM Tris buffer (pH 7.8) and 100 μM KCl were heated to 90 °C for 5 min, before cooling to room temperature at a rate of 1 °C/min. Oligos were mixed with 40 μM ThT and incubated at RT for 5 min. 25 μL of each oligonucleotide/ThT mixture was then transferred in triplicate to wells of a black walled 96 well plate (Greiner) containing 25 μL of a serial titration of quarfloxin or CX-5461. The plate was incubated for 30 min at 37 °C in the dark and analysed using a FLUO Star Omega Plate reader (BMG Labtech) at Ex 420 nm Em 480 nm.

### 2.11 Quarfloxin-staining of G4 agarose gel

10 μM oligos were folded as described above and incubated with 100 nM quarfloxin for 30 min. Samples were mixed with 60% v/v glycerol and loaded into a 1% TAE agarose gel, either mixed with 1× SYBR safe DNA dye (Invitrogen) or without dye and electrophoresed at 100 V for 20 min. Gels were imaged on an Azure 500 imager (Azure Biosystems) using 365 nm for quarfloxin and the epi-blue setting for SYBR-safe.

### 2.12 Immunofluorescence

Parasite-infected erythrocytes were smeared onto glass microscope slides and fixed for 15 min in 4% PFA in PBS. Slides were rinsed in PBS, permeabilised for 5 min in 0.1% Triton-x 100 in PBS, and then blocked for 1 h in blocking solution (3% w/v BSA in PBS). Mouse-BG4 antibody (Ab00174–1.1, AbsoluteAntibody) was diluted 1:250 in blocking solution and incubated overnight at 4 °C. Slides were rinsed 3 times in PBST and incubated in 1:1000 goat anti mouse Alexa fluor 546 for 1 h, then rinsed again and counterstained with 5 μg/mL DAPI. Slides were rinsed in dH_2_O then mounted with Diamond Prolong antifade (Invitrogen). Samples were imaged on a Zeiss LSM700 confocal, 60×, pinhole – 1 AU, 16 bit at 405 nm for DAPI and 532 nm for BG4 to generate representative images, and by Nikon Microphot SA microscopy (as above for TUNEL assay) using a 100× objective to generate signal intensity measurements from large numbers of cells. Signal intensity was calculated using ImageJ software.

### 2.13 Flow cytometry

The published BG-Flow protocol for mammalian cells (De Magis et al., 2021) was adapted for *Plasmodium* parasites. All centrifugation steps were performed at 500×*g* for 3 min using low brake. Synchronous parasitised erythrocytes were fixed in freshly made 4% PFA (w/v PBS) containing 0.0025% (v/v) glutaraldehyde in PBS for 30 min, rinsed for 5 min in PBS, then permeabilised in 0.05% Triton-x 100 (v/v) in PBS for 15 min. Samples were rinsed for 5 min in PBS then blocked in 3% BSA for 1 h at RT with rotation. Samples were incubated overnight at 4 °C with rotation in 1:250 BG4, followed by 1 h 1:1000 donkey anti-mouse IRDye 680RD (LI-COR). Samples were rinsed 3 × 5 min in PBS following each antibody change and costained in 5 μg/mL DAPI. Cells were read on an Attune NxT flow cytometer. DAPI signal was detected by 405 nm Violet laser (bandpass 440/50 nm) and BG4 by 637 nm Red (bandpass 720/30 nm) optical filters. For each sample 20,000 events were recorded, doublet discrimination performed by plotting forward scatter height versus area, and singlet population gated for +/+ DAPI/BG4 signal.

### 2.14 RNA-seq

Amplification free RNA-Seq was performed as a modified DAFTseq protocol (Chappell et al., 2020). Briefly, tightly synchronous trophozoite infected erythrocytes at 30–32 h.p.i were cultured with 2× EC_50_ of either quarfloxin, CX-5461, or no compound for 4 h. Parasites were saponin released and stored in Trizol reagent (Invitrogen). Total RNA was extracted as described previously (Kyes et al., 2007). Genomic DNA was removed using Turbo DNase clean-up kit (ThermoFisher) according to manufacturer's instructions and contamination with residual gDNA was checked by PCR amplification using primers designed across an intron of gene PF3D7_0708800 (Supplementary Table 4). PolyA mRNA was enriched by using NEB polyA module according to kit instructions. First strand cDNA was synthesised using Superscript IV kit (ThermoFisher). Each 20 μL reaction contained 5× FSS buffer, 0.4 μg/μL Oligo d(T) primers, 0.4 μg/μL random primers, 10 mM dNTPs, 0.5 μg/μL ActinomycinD, 0.1 M DTT, 1 μL Superscript IV reverse transcriptase, 20 U RNaseOUT, 9.5 μL mRNA. Samples were subjected to 25*°*C for 10 min, 42*°*C for 60 min, 4*°*C hold. cDNA was cleaned using Ampure XP beads. Second strand cDNA synthesis was performed using NEB Next Ultra II Second Strand Synthesis Module. Each reaction was performed in 50 μL containing 5 μL 10× second strand buffer and 4 μL enzyme mix. Synthesis was performed in a thermocycler at 16*°*C for 2.5 h and a second Ampure bead clean-up was performed. Following quality control check of cDNA, samples were then processed and sequenced at the Cambridge Genomic Services sequencing facility. Briefly, samples were sheared using a Covaris M220, libraries were prepared using KAPA HyperPrep kit (Roche) and samples were sequenced using Illumina NextSeq 550 MO. FASTQ files were accessed from Ilumina BaseSpace and processed for alignment.

### 2.15 RNA-seq data analysis

Paired end reads from FASTQ files were trimmed using Trimmomatic (v0.39) (Andrews S., 2010; Bolger et al., 2014) and aligned to *P. falciparum* 3D7 genome v58 (Aurrecoechea et al., 2009), using HISAT2 (v2.2.0) (Kim et al., 2019) and were indexed using BAMtools (Barnett et al., 2011). Reads were counted using featureCounts from the SubRead (v2.0.2) package (Liao et al., 2014). Differential expression was performed in RStudio with R (3.6.3) (R Core Team, 2018; RStudio Team, 2015) using DESeq2 (v1.36) with adjusted p value < 0.05 and log2FC > 1 (Love et al., 2014). Heatmaps and volcano plots were used to visualise data spread, and assess for noise across samples, following which outlier data was removed (Supplementary Fig. 7). Multiple R packages were used to manage and visualise data (Tidyverse, 2019; Wickham, 2009; Wickham and Henry, 2019). Gene ontology enrichment was performed using ReviGO (Supek et al., 2011).

### 2.16 Validation of RNA-seq by qRT-PCR

Follow-up validation was performed using primers designed to 6 differentially expressed genes as determined by RNA-Seq. Parasites were treated as above, extracted by phenol-chloroform, and cDNA synthesised using SensiFAST cDNA synthesis kit (Bioline). Reactions were performed in a QuantStudio 6 pro real-time PCR system (Applied Biosystems) using SensiFAST SYBR Lo-ROX kit (Bioline). Melt curve analysis was performed. Mean Ct values were calculated from 3 biological repeats and normalised against housekeeping genes PF3D7_0717700 (serine-tRNA ligase) and PF3D7_1444800 (aldolase) to find ΔΔCt. Fold change against the untreated control was calculated and plotted.

### 2.17 Analysis and statistical tests

Analysis of microscopy images and densitometry were performed in ImageJ unless otherwise stated (Schneider et al., 2012). Statistical analysis was performed in GraphPad Prism v8.

### 2.18 Data availability

RNA-SEQ data have been deposited at GEO, under accession number GSE235142.

## 3 Results

### 3.1 Quarfloxin is more toxic to *Plasmodium* parasites than CX-5461

**Fig. 1:**
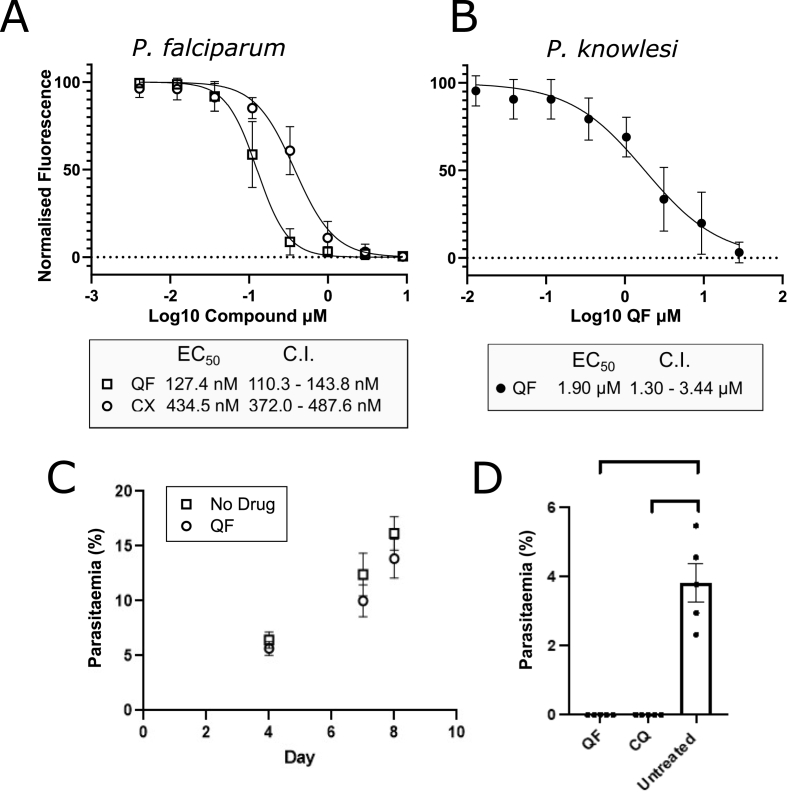
**Quarfloxin and CX-5461 are toxic to *Plasmodium* species**. A) Dose response curves of quarfloxin and CX-5461 in *P. falciparum* and B) Dose response curve for quarfloxin in *P. knowlesi*. Curves were generated from MSF assays (3 biological repeats) and used to calculate EC_50_ values. *P. falciparum* was more sensitive to quarfloxin (EC_50_ = 127.4; CI = 110.3–143.8 nM) than CX-5461 (EC_50_ = 434.5 nM; CI = 372.0–487.6 nM). *P. knowlesi* was less sensitive to both quarfloxin (EC_50_ = 1.90 μM; CI = 1.30–3.44 μM) and CX-5461 (which was limited by solubility, precluding the generation of a dose response curve at reasonable concentrations of the DMSO vehicle; highest dose tested was 25 μM). C) Results of a preliminary *in vivo* Peter's test using 0.6 mg/kg quarfloxin administered IV to P. *berghei-*infected mice (n = 5 per group). Despite a small reduction in parasite burden (p = 0.14) the mice did not clear infection over 8 days. D) Parasitaemia of mice (n = 5 per group) 5 days post infection with 10^5^ *P. berghei* parasites and then daily treatment with 25 mg/kg quarfloxin administered IP. Chloroquine (CQ) is shown as a positive control for parasite clearance.

The EC_50_ of CX-5461 in *P. falciparum* parasites (3D7 strain) was measured and compared with the EC_50_ of quarfloxin, which we previously reported at 110 nM (CI = 108.0–120.0 nM) (Harris et al., 2018). The EC_50_ measured here for quarfloxin was similar at 127.4 nM (CI 110.3–143.8 nM). The EC_50_ of CX-5461, measured using a standard 48 h Malaria SYBR-green Fluorescence (MSF) assay (Smilkstein et al., 2004), was 434.5 nM (CI = 372.0–487.6 nM), meaning that *P. falciparum* is fourfold more sensitive to quarfloxin than CX-5461 (Fig. 1A). We also evaluated the relative toxicity of these compounds in the other readily cultured human malaria species, *P. knowlesi (*Fig. 1B), since antimalarial drugs are particularly valuable if they work across species and *P. knowlesi* is often used as a model for the second major human parasite, *P. vivax*. While both CX-5461 (EC_50_ > 25 μM) and quarfloxin (EC_50_ 1.64 μM) were more than an order of magnitude less active against *P. knowlesi* than *P. falciparum*, this is in line with previous studies which have shown that *P. knowlesi* is less sensitive to many other antimalarials than *P. falciparum* (van Schalkwyk et al., 2019). Satisfactory dose response curves could not be generated for CX-5461 in *P. knowlesi* without either exceeding the solubility of the drug or unacceptable levels of DMSO vehicle control (>0.5% DMSO final volume, maximum concentration tested was 25 μM).

The more potent of the compounds, quarfloxin, was then evaluated in an *in vivo* murine model against the model parasite species *P. berghei.* An initial Peter's test treated infected mice at doses of 100× of the EC_50_ measured for *P. falciparum* (0.6 mg/kg). The compound was administered through the intravenous route. Quarfloxin-dosed mice showed a slight decrease in parasite burden, as determined by tail-vein smears (Fig. 1C), but there was no significant efficacy. The dose was then increased to 25 mg/kg with delivery by intraperitoneal injection (Fig. 1D). Previous patent documents for quarfloxin had suggested that the compound was well-tolerated in mice at this dosage (Whitten et al., 2006), and under these conditions, mice completely cleared all blood-stage parasitaemia at day 5 post-infection, as determined by examination of peripheral tail-vein smears. However, we observed severe adverse effects, both via the intra-peritoneal route (necessitating euthanasia 5 days post infection) and via the intravenous route (causing severe inflammation at the injection site). Further experiments were therefore precluded, as it was clear that the tolerance reported in the patent was not reproduced here, and that more extensive *in vivo* titration experiments would be required, which were beyond the scope of this study and the availability of the compound.

### 3.2 *P. falciparum* cannot readily develop resistance to quarfloxin in *invitro* culture

Developing resistance via prolonged exposure to drug candidates may elucidate crucial ways in which the compound interacts with the parasite, providing mechanistic insights. We therefore tried to develop quarfloxin-resistant parasites via long term *in vitro* culture of *P. falciparum* with continuous exposure to sub-lethal levels of quarfloxin, slowly increasing in concentration. After nine months, parasite populations were obtained that were able to grow on ∼2× EC50 quarfloxin; however MSF analysis of two clones (C2-1 and C2-5) that were generated under continual quarfloxin pressure showed no significant difference in quarfloxin EC50 compared to wildtype 3D7 parasites (Supplementary Fig. 1). Removing quarfloxin selection pressure for a short-term period (∼one week) removed acquired tolerance (data not shown). Together this indicated that tolerance was not genetically driven. This inability to generate quarfloxin-resistant parasites meant that it was not possible to define a mechanism of action for the compound by identifying mutated resistance genes, an approach commonly used in *Plasmodium* target identification (Cowell et al., 2018).

### 3.3 Quarfloxin is distributed throughout *P. falciparum* parasite cells

The subcellular location of a drug candidate can potentially help to reveal its mechanism of action. For example, chloroquine accumulates in the food vacuole (Yayon et al., 1985) – the target organelle in which it inhibits haem detoxification. Therefore, the fluorescent nature of quarfloxin as a fluoroquinolone was exploited to assess its localisation within the parasite cell using live fluorescence microscopy. (Equivalent experiments with CX-5461 were precluded by its non-fluorescent nature, which was confirmed in-house.) Quarfloxin has an excitation maximum of approximately 350 nm and emission maximum of 550 nm (Drygin et al., 2009). In conjunction with live-cell-compatible dyes emitting in the far-red range (DRAQ5, MitoTracker FR and Lysotracker FR), microscopy on live parasites was used to assess any compartmentalisation of the compound to the nucleus, mitochondrion or food vacuole.

**Fig. 2:**
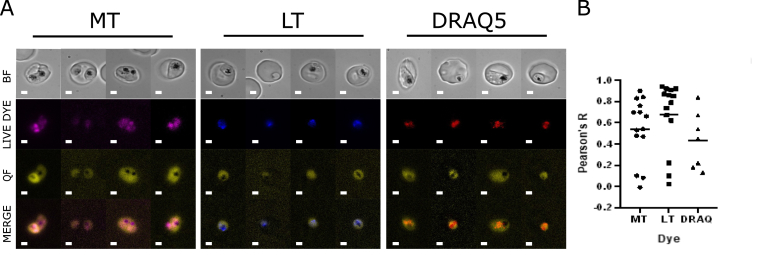
**Localisation of quarfloxin within cells**. A) Representative images of live parasites stained with quarfloxin (yellow) and MitoTracker (magenta, mitochondrial signal); LysoTracker (blue, food vacuole signal); or DRAQ5 (red, nuclear signal) live-cell-compatible dyes. Quarfloxin fluorescence was not compatible with cell fixation, hence all microscopy was on live cells. Scale bar = 2 μm. B) Images were analysed for colocalisation of quarfloxin and organellar signal using ImageJ (n = ∼10 cells). A broad range of Pearson's R scores suggest no specific association with individual organelles. (For interpretation of the references to colour in this figure legend, the reader is referred to the Web version of this article.)

Quarfloxin preferentially localised within infected erythrocytes over empty erythrocytes (Supplementary Fig. 2) and was confined within the parasite membrane in infected erythrocytes (Fig. 2). However, there was no specific intracellular concentration. Quarfloxin signal was generally seen throughout the cell, hence it appeared in nuclei (as anticipated) (Fig. 2A, red), but also around the mitochondria (Fig. 2A, magenta) and the food vacuole (Fig. 2A, blue). Colocalisation analysis confirmed that there was no particularly strong association with any specific organelle tested (Fig. 2B). Signal was highly diffuse across the entire parasite cell (being excluded only from the inorganic haemozoin crystal in mature parasites) and it did not appear in foci.

### 3.4 CX-5461 does not target P. falciparum through topoisomerase inhibition

CX-5461, like quarfloxin, was originally thought to function as an RNA polymerase I inhibitor in human cells (Haddach et al., 2012). Our earlier work already ruled out this mode of action for quarfloxin in *P. falciparum*, probably due to the non-canonical arrangement of rRNA genes in this parasite (Harris et al., 2018). More recently, however, it was reported that the primary mechanism of action for CX-5461 in human cells is actually to cause DNA damage through topoisomerase II inhibition (Bruno et al., 2020; Pan et al., 2021). Accordingly, in cancer cells, resistance to topoisomerase inhibitors tracks with resistance to CX-5461 (Bruno et al., 2020). This is a potential mechanism for CX-5461 in *P. falciparum*. It is known the parasite encodes well-conserved topoisomerases (Chowdhury and Majumder, 2019) and is sensitive to topoisomerase poisons such as etoposide (Dumetz et al., 2021).

We therefore evaluated the hypothesis that CX-5461 could affect *Plasmodium* topoisomerase activity, utilising an existing line with an altered capacity for DNA double-strand-break repair. This line was generated via a mutated *RAD54* gene (Dumetz et al., 2021). The *P. falciparum RAD54* gene happens to encode an rG4 that reduces gene translation, so an rG4-mutant produces far more RAD54 protein than a wildtype parasite. Accordingly, the mutant line has reduced sensitivity to etoposide, presumably through more efficient DNA repair (Dumetz et al., 2021). If CX-5461 acts similarly to etoposide, this mutant should also have reduced sensitivity to CX-5461. However, we found negligible change in the EC_50_ of CX-5461 in ‘RAD54-high’ parasites, in contrast to their differential etoposide sensitivity. Therefore, we could not generate positive evidence for this mode of action in *Plasmodium*.

### 3.5 DNA damage phenotypes are caused in *P. falciparum* by both quarfloxin and CX-5461

Despite the negative data on topoisomerase inhibition, it remained possible that quarfloxin and CX-5461 would cause DNA damage in *P. falciparum* by an alternative route, such as stabilising G4s and hence stalling DNA replication forks. Experiments were therefore undertaken to explore the ability of both compounds to cause DNA damage in *P. falciparum*.

**Fig. 3:**
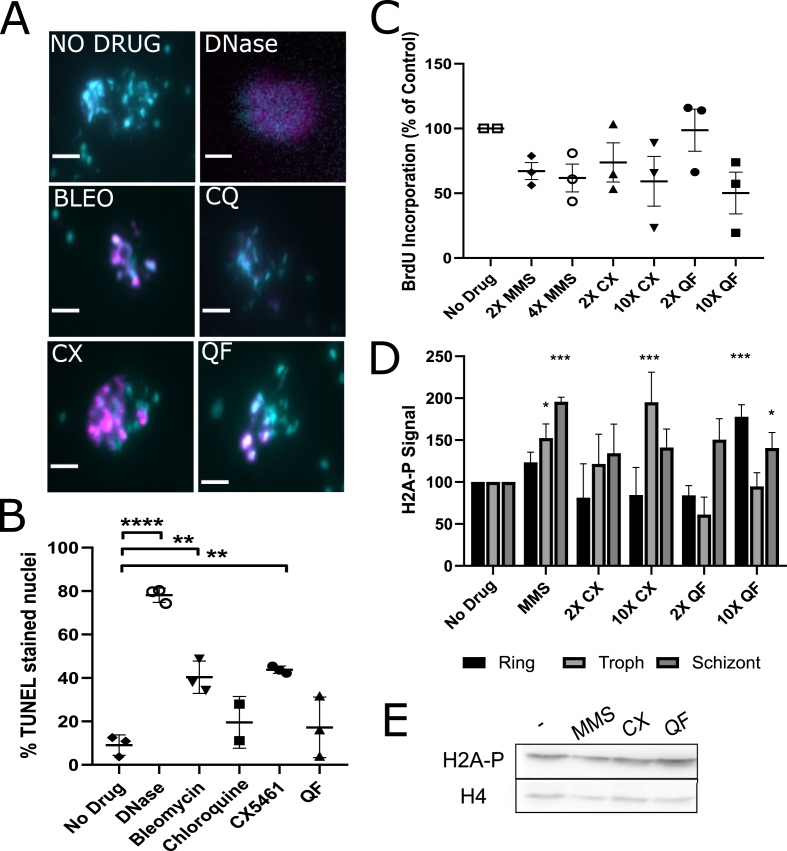
**Quarfloxin and CX-5461 are associated with DNA damage**. A) TUNEL staining of compound treated parasites. Mixed-stage cultures were incubated with compounds (2× EC_50_) for 4 h, stained with TUNEL kit (magenta), counterstained with DAPI (cyan) and imaged via light microscopy (Scale bar = 2 μm). DNase treatment and bleomycin were used as positive controls, and chloroquine (a known antimalarial that does not target DNA directly), as a negative control. B) Nuclei were counted and the percentages of nuclei co-stained with TUNEL and DAPI were calculated and plotted (3 replicates: n = 100 cells). Data were tested for significant differences against the untreated control via one way ANOVA with Dunnett's posthoc and were not significant. C) BrdU incorporation measured by ELISA. Parasites were incubated with high and low concentrations of compound (10× or 2× EC_50_) or MMS control (4× or 2× EC_50_; EC_50_ = 92 μM) for 1 h and pulsed with BrdU for a following 1 h prior to ELISA assay. BrdU incorporation was measured as signal intensity as a percentage of signal in untreated controls. Three biological repeats were assessed for significant differences by one way ANOVA. D) H2A-P signal intensity as measured by densitometry on three biological repeats of western blots after parasites had been treated with 2× EC_50_ quarfloxin, CX-5461 or MMS for 2 h. 2-Way ANOVA with Dunnett's posthoc was performed. E) Representative Western blot showing H2A-P signal; loading was controlled with histone H4 band density. All stars indicated significance at × P < 0.05, **P < 0.01 ***P < 0.001, P < 0.0001. (For interpretation of the references to colour in this figure legend, the reader is referred to the Web version of this article.)

DNA strand breakage was assessed by terminal deoxynucleotidyl transferase dUTP nick end labelling (TUNEL) assay (Fig. 3A–B). In this assay, broken or nicked DNA ends are labelled with a nucleoside that can then be imaged using fluorescence microscopy. Representative images (Fig. 3A) showed some colocalisation of TUNEL and DAPI (DNA) stain in control cells, but this occurred in significantly more cells after treatment with CX-5461 (p = 0.0011), as well as after treatment with two positive controls, bleomycin (p = 0.0025) and DNase (p < 0.0001), which cause double and single stranded DNA breaks. This indicated a causative relationship between all these compounds and DNA damage (Fig. 3B). Quarfloxin-treated samples also had more co-stained nuclei than controls, but fewer than CX-5461-treated samples: TUNEL staining here was at a level similar to that in parasites treated with chloroquine, an antimalarial that does not damage DNA as its primary mechanism of action. Accordingly, neither chloroquine nor quarfloxin showed a statistically significant increase in TUNEL staining over the control.

Checkpoint responses to DNA damage are not well-characterised in *Plasmodium*, but in most eukaryotic cells, damage triggers a reduction in DNA replication – either directly by impeding replication forks, or via checkpoint responses that prevent S-phase entry (a G1-S checkpoint) or reduce the progress of an ongoing S-phase (an intra-S-phase checkpoint) (Chatterjee and Walker, 2017). Therefore, DNA replication was measured after treatment with quarfloxin and CX-5461, using nascent DNA labelling (Fig. 3C). Parasites made to express a viral thymidine kinase enzyme can scavenge pyrimidine nucleosides during replication, and thus nucleoside labelling techniques, which are otherwise unavailable in *Plasmodium*, can be leveraged to quantify nascent DNA replication (Merrick, 2015). Here, the nucleoside analogue bromodeoxyuridine (BrdU) was used to label synchronous trophozoite parasites (this being the cell-cycle stage in which S-phase occurs) for 1 h directly after a 1 h pulse of drug. We used high (10× EC_50_) and low (2× EC_50_) concentrations of quarfloxin or CX-5461, alongside a control DNA-damaging agent, methylmethanesuphonate (MMS), at 2× and 4× EC_50_ levels (EC_50_ = 92 μM). MMS is well-characterised in model cells to alkylate DNA and thus acutely arrest ongoing replication forks (Chang et al., 2002; Merrick et al., 2004). Nascent DNA synthesis was measured by BrdU ELISA (Merrick, 2015). DNA replication trended downwards after DNA damage with MMS and similar trends were observed for quarfloxin and CX-5461, albeit this was not statistically significant. This response was more pronounced in CX-5461, where both levels of compound reduced DNA replication; quarfloxin required higher levels (10× EC_50_) to achieve similar disruption of replication.

The DNA damage response in many eukaryotic cells is characterised by phosphorylation of the H2A.X histone (Mah et al., 2010). *P. falciparum* lacks H2A.X but was recently reported to make an analogous DNA damage response by phosphorylation of H2A (Goyal et al., 2021). This can be observed using an antibody to phosphorylated human H2A.X, which is cross-reactive against phosphorylated *P. falciparum* H2A (Fig. 3D and E). Western blot experiments were performed in triplicate on lysates collected from synchronised ring, trophozoite and schizont parasite cultures after 2 h compound treatments, to assess if there were stage-specific differences in DNA damage signalling via H2A-P. The signal appeared to be both stage dependent and dose dependent. MMS and CX-5461 both elicited phosphorylation primarily in trophozoites and schizonts, consistent with DNA damage occurring in a replication-linked fashion. Quarfloxin gave less consistent results but at higher levels it elicited the signal in rings and schizonts but not, unexpectedly, in trophozoites. In all cases, the increase in H2A-P signal over the background level seen in undamaged cells was modest (under 2-fold) and also transient, peaking within ∼2 h (data not shown).

### 3.6 QF and CX-5461 bind *in vitro* to *P. falciparum* G-quadruplexes

The data in Fig. 3 raised the question of exactly how DNA damage is caused by quarfloxin and CX-5461. Supplementary Fig. 3 has already showed that it is unlikely to be primarily via topoisomerase inhibition. However, since these compounds are designed to bind to G4s, they could hypothetically stabilize such structures, impede DNA replication forks and thus cause fork stalling and DNA breakage (David et al., 2016; Moruno-Manchon et al., 2017; Paeschke et al., 2011). There is no direct evidence for this as yet in *P. falciparum.*

**Fig. 4:**
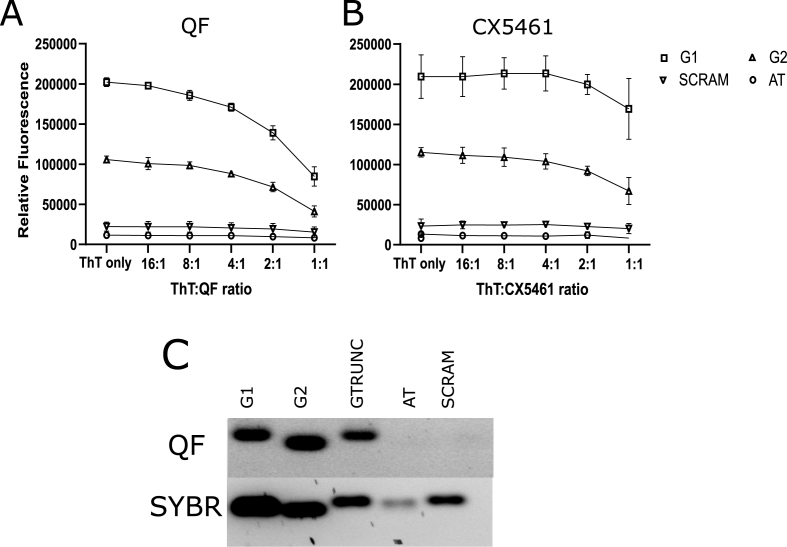
**Quarfloxin and CX-5461 can bind to G4 sequences found in *P. falciparum***. Oligo sequences were allowed to fold into G4s by annealing in the presence of monovalent cations and then incubated in the presence of 40 μM ThT and a dilution gradient of either A) quarfloxin or B) CX-5461. Oligos were incubated for 30 min before reading the ThT fluorescence as relative fluorescence units (RFU). Average RFUs were calculated from three blank-corrected biological repeats. Oligo sequences were G4-encoding (G1, G2), scrambled (scram) or A/T-only (AT). C) Visualisation of quarfloxin binding by agarose gel electrophoresis. 10 μM oligos incubated with 100 nM quarfloxin were imaged under UV (for quarfloxin) and blue light (for SYBR green).

Oligonucleotides encoding *P. falciparum* G4 sequences were used to assess the G4 binding capacity of quarfloxin and CX-5461 *in vitro* (Fig. 4). Oligos were folded *in vitro* and their correct folding was confirmed by incubation with Thioflavin T (ThT), a small molecule that fluoresces only when bound to G4 structures (De La Faverie et al., 2014). Two different G4 structures were selected, alongside controls with a scrambled sequence, or a sequence of A/T-only, neither of which bound to ThT (Fig. 4A and B). A titration of either quarfloxin or CX-5461 was added to the ThT-bound oligos to test whether these compounds could bind to G4s and outcompete ThT, resulting in a ‘quenched’ signal. Both quarfloxin (Fig. 4A) and CX-5461 (Fig. 4B) could compete with ThT, even at lower-than-1:1molar ratios of ThT:drug.

Due to its fluorescent property, the binding of quarfloxin to G4 folded oligos could also be visualised on an agarose gel (Fig. 4C). Incubation of 10 μM folded oligo with 100 nM quarfloxin showed preferential binding to the G4 folded oligos. A truncated oligo bound more weakly (possibly by forming inter-molecular G4s) and scrambled or A/T-only oligos did not bind at all.

### 3.7 QF and CX-5461 do not detectably stabilize *P. falciparum* G-quadruplexes *in cellulo*

**Fig. 5:**
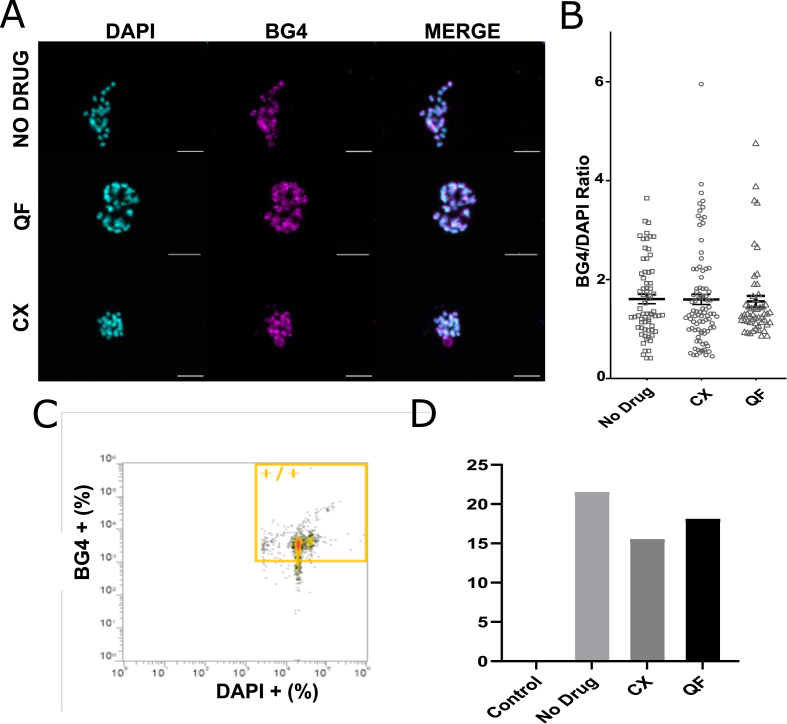
**Quarfloxin and CX-5461 do not alter the total G4 signal detected *in cellulo****.* A) *P. falciparum* cultures were incubated in no drug, 2× EC_50_ quarfloxin or CX-5461 for 4 h prior to analysis by immunofluorescence. Cells were stained with DAPI (cyan) and BG4 (magenta) and imaged by confocal microscopy. Scale bar = 5 μm. B) Signal intensity per cell was measured in ImageJ and represented as BG4 intensity relative to DAPI signal (n = ∼50 cells). C) Flow cytometry analysis was performed, recording 20,000 events, gated for singlets, and then for +/+ BG4/DAPI signal. Example data are shown from cells treated with CX-5461. D) The percentage of BG4-positive cells in a population of 20,000 is shown after treatment with quarfloxin, CX-5461 or no drug. (For interpretation of the references to colour in this figure legend, the reader is referred to the Web version of this article.)

The data in Fig. 4 showed that both compounds can bind to parasite derived G4s *in vitro,* but not that they actually do so *in vivo.* The BG4 antibody (Biffi et al., 2013) has been used to visualise G4s in fixed cells in immunofluorescence assays (Biffi et al., 2014; Craven et al., 2021; Wang et al., 2019), including in *P. falciparum* (Harris et al., 2018), and more recently in flow cytometry (De Magis et al., 2021). Cultured parasites were incubated with quarfloxin or CX-5461 and analysed by immunofluorescence (Fig. 5A). BG4 signal was present primarily in nuclei, as previously reported (Harris et al., 2018), suggesting that the signal is more representative of DNA G4s than of RNA G4s – indeed, the great majority of PQSs in the *P. falciparum* genome are found in telomere repeats. Quarfloxin and CX-5461 did not significantly affect the BG4 signal intensity (Fig. 5B). Parasites were also assessed by flow cytometry for a more quantitative measurement (Fig. 5C), but again no significant difference was observed (Fig. 5D).

We previously generated mutants in two *P. falciparum* RecQ helicases, termed *Pf*BLM and *Pf*WRN. These helicases play key roles in unwinding DNA G4s, and the mutant lines showed increased sensitivity to the G4-stabilising agent TMPyP4, as expected if they were impaired in their ability to unwind drug-stabilised G4s (Claessens et al., 2018). The same parasite lines were tested here, but only a small, non-significant shift in EC_50_ was observed (Supplementary Fig. 4, EC_50_ values spanned 125.3–149.3 nM, lower and upper CI limits were 111.2–184.1 nM). Collectively, although quarfloxin and CX-5461 could clearly bind to G4 structures *in vitro*, the evidence was inconclusive for any broad G4-stabilising effect *in cellulo.*

### 3.8 QF and CX-5461 have acute effects on the *P. falciparum* transcriptome

Since it was not possible, at a whole-cell level, to detect any bulk effects of quarfloxin or CX-5461 on *Plasmodium* G4s, we sought to improve our resolution and detect any gene-by-gene effects by exploring the potential of these compounds to affect the transcriptome. Transcriptome-wide RNA-Seq, optimised for *P. falciparum* (Chappell et al., 2020), was performed on trophozoite parasites after treatment with no drug, quarfloxin or CX-5461 at 2× EC50 for 4 h, between 30-32 h and 34–36 h post invasion (h.p.i). At this stage, the parasites should be performing S-phase. A moderate level and short period of treatment were chosen to capture only the most acute drug-specific effects, rather than any general changes associated with parasite death.

**Fig. 6:**
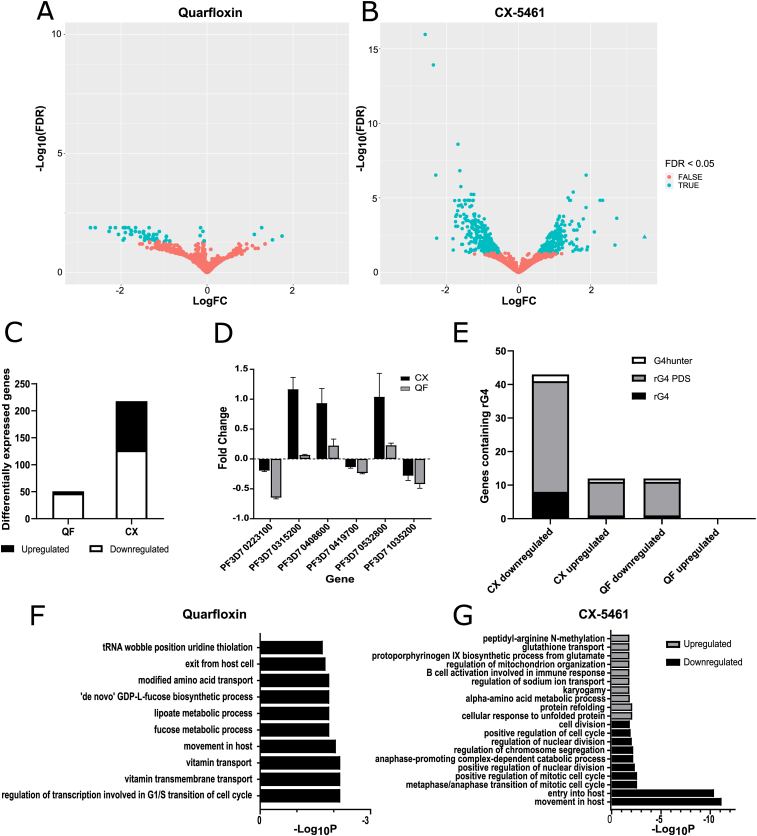
**Quarfloxin and CX-5461 dysregulate the parasite transcriptome**. A,B) Volcano plots of differentially expressed (DE) genes (adj. P < 0.05, log2FC > 1) for A) quarfloxin and B) CX-5461 compared to untreated control. C) Bar chart of total numbers of up- and down-regulated DE genes after each compound treatment. D) Validation of RNA-Seq by qRT-PCR on six selected genes after parasites received the same treatment with quarfloxin or CX-5461. The qRT-PCR experiments were repeated in biological triplicate and expression of each gene was calculated versus the average of two housekeeping genes. E) Bar chart of the number of genes dysregulated by either compound that also contained rG4s (generated by comparison with a published rG4-seq dataset of rG4s detected in the presence or absence of pyridostatin) or DNA G4s (by comparison with published predictions from G4Hunter). F, G) GO enrichment analysis was performed. Top 10 biological processes amongst DE genes are plotted for F) quarfloxin downregulated DE genes and G) CX-5461 up-and down-regulated DE genes.

Both compounds caused significant transcriptional deregulation (Fig. 6A–B) although greater numbers of genes were differentially expressed (with a fold-change >2, FDR <0.05) after CX-5461 (218 genes, Fig. 6B) than after quarfloxin (51 genes, Fig. 6A) (Fig. 6C, Supplementary Tables 1–2). Quarfloxin elicited very little upregulation, but downregulation of multiple genes encoding ncRNAs, including some ribosomal RNA fragments, and *PIR* genes, a family of sub-telomeric genes associated with virulence. Downregulated genes in CX-5461 included several ncRNAs, including some ribosomal RNAs, but again without a strong, comprehensive effect suggestive of RNA polymerase I inhibition). Other downregulated genes included rhoptry and apical-complex associated genes, and merozoite surface protein (MSP) encoding genes. Upregulated genes included *PIR*s (in direct contrast to the downregulatory effect of quarfloxin), and heatshock associated genes (Supplementary Table 2). Overall, genes were more likely to be downregulated than upregulated by both compounds. Around 34% of genes modulated by CX-5461 had unknown functions, and ∼25% for quarfloxin, which is not unusual in the *Plasmodium* genome.

To further validate the RNA-Seq data, qRT-PCR was performed on a small set of 6 modulated genes, selected because they had large fold changes (so would be easier to detect by qRT-PCR) or had low P values (potentially more biologically relevant). All but one of these genes (PF3D7_0315200 for quarfloxin) displayed a positive or negative fold change concordant with the RNA-Seq data (Fig. 6D).

We assessed the G4 content of deregulated genes using several published datasets of genes predicted to encode G4s (Dumetz et al., 2021; Gage and Merrick, 2020). Concerning RNA G4s, 10 quarfloxin-modulated genes and 43 CX-5461-modulated genes encoded putative rG4s (Fig. 6E, Supplementary Table 3) (Dumetz et al., 2021). The rG4-encoding genes were largely downregulated. They accounted for 19.6% of all quarfloxin-modulated genes and 19.7% of CX-5461-modulated genes; however, these proportions are similar to the proportion of all *P. falciparum* genes that encode putative rG4s when stabilised by the compound pyridostatin (22%), so there was no clear enrichment of rG4-encoding genes. By comparison, only a few dysregulated genes contained PQSs in their DNA sequence, as predicted by G4Hunter (Gage and Merrick, 2020) (Supplementary Table 3).

In addition to directly regulating G4-encoding genes, it was possible that quarfloxin and CX-5461 would ‘indirectly’ affect particular transcriptional pathways, as the parasites made an acute response to stress. Gene ontology (GO) enrichment analysis was performed using ReviGO (Supek et al., 2011) to detect any enriched gene activities amongst the dysregulated genes (Fig. 6F–G, Supplementary Fig. 5). Quarfloxin caused very little upregulation of genes and so analysis was performed on downregulated genes only. Quarfloxin treatment was associated with downregulation of processes related to vitamin transport, mitotic cell cycle transcription and movement in the host (Fig. 6F). Enriched molecular functions included peroxidase and sulfotransferase activity, DNA exonuclease activity and NAD dependent histone deacetylation (Supplementary Fig. 5A). These pathways, however, were generally represented by only small numbers of genes. CX-5461 treatment was characterised by downregulation in biological processes related to cell cycle regulation, nuclear division, mitotic processes, and parasite entry into the host (Fig. 6G). Further enrichment was observed in GO terms related to cell surface and NAD^+^ binding, peroxidase activity, and transmembrane transport (Supplementary Fig. 5B). Upregulated genes from CX-5461 treatment were enriched for GO terms associated with protein refolding, misfolded protein rebinding, programmed cell death and ion transport regulation (Fig. 6G, Supplementary Fig. 5C).

We performed further GO enrichment analysis on a larger group of genes showing a less-stringent fold change of 1.5 rather than 2 (Supplementary Fig. 6). This did not have any major effect on the GO terms for genes downregulated by quarfloxin or CX-5461, but it did seem to increase diversity of terms in genes that were upregulated following CX-5461 treatment. There was a notable increase in terms pertaining to ribosomal biogenesis and RNA transcription initiation compared to more stringent 2-fold expression analysis.

## 4 Discussion

This work explored the mode of action of two G4-binding compounds – previously developed and tested as anticancer agents in humans – for a further understanding of *Plasmodium* G4 interactions, and to establish a molecular mechanism of action for these agents in killing malaria parasites. We found that although quarfloxin and CX-5461 may be unsuitable as an initial drug-discovery avenue in their current form, they do provide insights into the activity of such compounds in *Plasmodium*, which is a major human pathogen and also an unusual, early diverging eukaryote with a guanine-poor genome.

We previously reported that quarfloxin had fast and potent parasite-killing activity *in vitro* (Harris et al., 2018). Here we showed that it is also active *in vivo* in a mouse model. This provides important information about the potential activity (and toxicity) of this compound, but high quarfloxin treatments severely debilitated the mouse host as well as the parasite. Although the original patent reported tolerance in mice (50 mg/kg and 25 mg/kg IV administration (Whitten et al., 2006)), this compound evidently has off-target toxicity in mice. Our data, for the first time, explore the viability of a G4 targeting compound as an antimalarial *in vivo*, but analogues with lower toxicity would be needed if this class of compounds was to be pursued further. When we tested the more recently developed CX-5461, which is still undergoing human cancer trials and is adequately tolerated in humans (Yan et al., 2021), we found this to be 4-fold less potent against *Plasmodium* than quarfloxin *in vitro*. This may not translate to *in vivo* activity, but without publicly available pharmacokinetic data, it was beyond the scope of the project to generate such data.

Quarfloxin killed the zoonotic *Plasmodium* species *P. knowlesi* as well as *P. falciparum*, and its apparently lower potency in *P. knowlesi* may be a technical artefact, because *P. knowlesi* is cultured in high concentrations of horse serum that reduce the effective concentration of many drugs (van Schalkwyk et al., 2017, 2019). Alternatively, this may highlight a genuine difference in the biology of the two species: *P. knowlesi* has a G/C-balanced genome and hence a higher density of PQSs than *P. falciparum* (Gage and Merrick, 2020; Stanton et al., 2016). Perhaps, counterintuitively, this renders parasites less sensitive, rather than more sensitive, to G4 binding, if indeed G4 binding is central to the antimalarial activity. To pursue this issue, a whole range of G4-binding compounds of various classes would need to be tested in *P. knowlesi.* (Human cells notably have a very high density of PQSs, yet quarfloxin is less potent against human cells (Kerry et al., 2017; Harris et al., 2018) than *P. falciparum.* There are, however, many possible reasons for this, such as the different arrangement of rRNA genes in human cells, or DNA damage responses being more robust in human cells. Quarfloxin is similarly more potent *in vitro* against another single-celled protozoan, *T. brucei*, than it is against cultured human cells (Kerry et al., 2017).)

In *P. falciparum* both compounds had steep dose response curves: quarfloxin in particular had a steep curve characterised by high *m* values, indicating greater potency in small increments above the EC_50_. This hallmark was present across species for both compounds, although to a greater extent in quarfloxin. Published work has shown that two compounds with the same EC_50_ value but differing *m* values can have different inhibitory effects, with a steeper slope associated with better inhibitory effects at clinical levels (Shen et al., 2008).

Resistance can potentially be recapitulated in culture, which is useful for identifying mutated gene targets by sequencing resistant parasites (Corey et al., 2016; Cowell et al., 2018). In clinical settings this has historically sometimes occurred within months of use of a new antimalarial drug (Capela et al., 2019; Ventola, 2015). Single enzyme targets can be most problematic in this regard, whereas ‘irresistible’ candidates are thought to have complex/multifactorial modes of action. Quarfloxin, however, proved ‘irresistible’ in culture, suggesting that it may have a complex mode of action and also precluding any identification of target(s) from resistant parasites. Similarly, the ubiquitous accumulation of quarfloxin within parasite cells, across all organelles examined, added no clues as to its site of action.

Since all these agnostic approaches could not elucidate how quarfloxin kills *Plasmodium*, we turned to hypothesis-driven experiments, based around the induction of DNA damage. DNA damage or replication stress may be a particularly effective antimalarial strategy because *Plasmodium* replicates fast and extensively during schizogony, and because it lacks the nonhomologous end-joining pathway for DNA repair and is entirely reliant on homologous recombination (Kirkman et al., 2014; Matthews et al., 2018; Roy et al., 2014). Indeed, several antifolate drugs that are effective in parasite clearance have been shown to inhibit DNA replication (Matthews et al., 2018).

Quarfloxin and CX-5461 could theoretically cause DNA damage either via topoisomerase II (as evidenced for CX-5461 in human cells (Bruno et al., 2020)), or via G4 stabilization and replication fork stalling (evidenced for several G4-binding compounds in model systems (Linke et al., 2021; Moruno-Manchon et al., 2017; Sun et al., 2019)). We found little consistent evidence for the former: although our experiments did not categorically rule it out, they at least showed that these compounds do not act exactly like etoposide. We did, however, find evidence for the latter: both candidates induced some level of DNA breakage; both induced phosphorylation of a damage-responsive histone and CX-5461 treatment was associated with increased stand breakage as evidence by TUNEL staining. Responses occurred fast – within 1–2 h of compound treatment – making it likely that they were compound-associated effects, not merely side-effects of cell death. All responses were stronger with CX-5461 than with quarfloxin (despite CX-5461 having no demonstrable effect through topoisomerase II). Hence, we concluded that CX-5461 may kill parasites primarily through DNA damage, but that quarfloxin-interacting pathways could be more complex, since parasites are more sensitive to quarfloxin than CX-5461, yet it is less DNA-damaging.

Pursuing this mechanism, we showed that quarfloxin and CX-5461 can bind *in vitro* to *Plasmodium* G4 sequences, displacing ThT (which is an end-stacking molecule – so quarfloxin and CX-5461 probably also end-stack on G4s, given the competitive nature of the assay). *In cellulo*, however, we detected no global increase of G4s in drug-treated parasites by antibody-based immunofluorescence or flow cytometry. In fact, the antibody-detectable G4 signal in *P. falciparum* is probably heavily dominated by G-rich telomeres, and these may not be the relevant target of these compounds, since we previously showed that sub-lethal quarfloxin has no effect on telomere maintenance (Harris et al., 2018).

Finally, we used RNA-Seq to identify gene-specific targets of the two compounds, hypothesising that a) genes encoding G4s that are directly bound by the compounds could be repressed, and b) genes in any stress pathways acutely affected would hint at the type of stress induced. We used a short, relatively low-level treatment to avoid non-specific effects, and accordingly found relatively few deregulated genes. (In a single precedent for this type of experiment, pyridostatin affected ten times as many genes, but parasites were exposed for an entire 48 h cycle (Gazanion et al., 2020). In a more comparable experiment, etoposide treatment for 6 h deregulated only 225 genes (Gupta et al., 2016).)

Among the genes affected by quarfloxin or CX-5461, only a minority encoded G4s – primarily RNA rather than DNA G4s. Our protocol would only detect transcripts that are turned over within 4 h, but with this caveat, neither compound seems to kill parasites mainly through binding to a large cohort of essential G4-encoding genes and impeding their transcription or inducing transcript degradation. Nevertheless, the affected G4-encoding genes were frequently affected by *both* compounds, supporting G4 binding as one mode of action for these compounds, and suggesting that where they do bind G4s, they bind to similar structures. The majority of these G4-encoding genes were also stabilised by pyridostatin in our earlier rG4-seq experiment (Dumetz et al., 2021), further indicating that dysregulation was probably driven by rG4 binding. The particularly low representation of PQSs at the DNA level, as predicted by G4Hunter even at a low stringency threshold, probably reflects the fact that *P. falciparum* encodes relatively few canonical G4s, whereas it encodes many more non-canonical motifs that can fold in RNA (as detected by rG4-Seq) but probably not in DNA.

RNA-Seq primarily detected early downstream effects of compound treatment: for CX-5461, a downregulation of replication and invasion pathways, consistent with the parasites detecting DNA damage and arresting their cell cycle and the maturation of daughter cells until damage can be repaired. This was accompanied by upregulation of heatshock pathways – a parallel with the mechanistic response to the frontline antimalarial artemisinin. Artemisinin causes broad-spectrum damage to both proteins and DNA (Gupta et al., 2016) and elicits a transcriptional response similar to the heatshock/misfolded protein response (Zhang et al., 2021). For quarfloxin, the same pathways were not affected: overall we saw fewer deregulated genes and they did not point clearly to a route to parasite killing, further suggesting a multifactorial mechanism of action. We then relaxed the fold-change threshold to 1.5 and repeated the GO enrichment analysis to capture any pathways affected at lower levels, but the enriched pathways did not change greatly. Interestingly, a secondary enrichment appeared in the CX-5461-upregulated genes, pertaining to RNA transcription initiation and associated processes.

Overall, even this detailed analysis at the cellular, genomic and transcriptomic levels did not reveal a single clear mode of action for quarfloxin in *P. falciparum.* We found some evidence for DNA damage, but not primarily through topoisomerase inhibition, widespread G4-stabilization or telomere dysregulation. The high antimalarial potency of quarfloxin, which was seen both *in vitro* in cultured parasites and *in vivo* in mouse models, is probably due to polypharmacology: a positive feature for an antimalarial drug, but one that nevertheless remains mysterious.

## FUNDING

This work was supported by a seedcorn grant from the Rosetrees Trust (M857), matched by the University of Cambridge Molteno fund, and a European Research Council grant ‘Plasmocycle’ (725126) to C.J.M. A.M.B is supported by the MRC [MR/N00227×/1 and MR/W025701/1], Sir Isaac Newton Trust, Alborada Fund, Wellcome Trust ISSF and University of Cambridge JRG Scheme, GHIT, Rosetrees Trust (G109130) and the Royal Society (RGS/R1/201,293) (IEC/R3/19,302).

## Declaration of competing interest

NONE.
