# Supplementary material for: Effects of the G-quadruplex-binding drugs quarfloxin and CX-5461 on the malaria parasite Plasmodium falciparum
Source: Int J Parasitol Drugs Drug Resist. 2023 Nov 24;23:106–19. doi: 10.1016/j.ijpddr.2023.11.007 (PMC10711401; doi:10.1016/j.ijpddr.2023.11.007)
Supplement: Multimedia component 2 [file mmc2.pdf]

Effects of the G-quadruplex-binding drugs Quarfloxin and CX-5461 on the malaria parasite *Plasmodium falciparum*

Supplementary figures

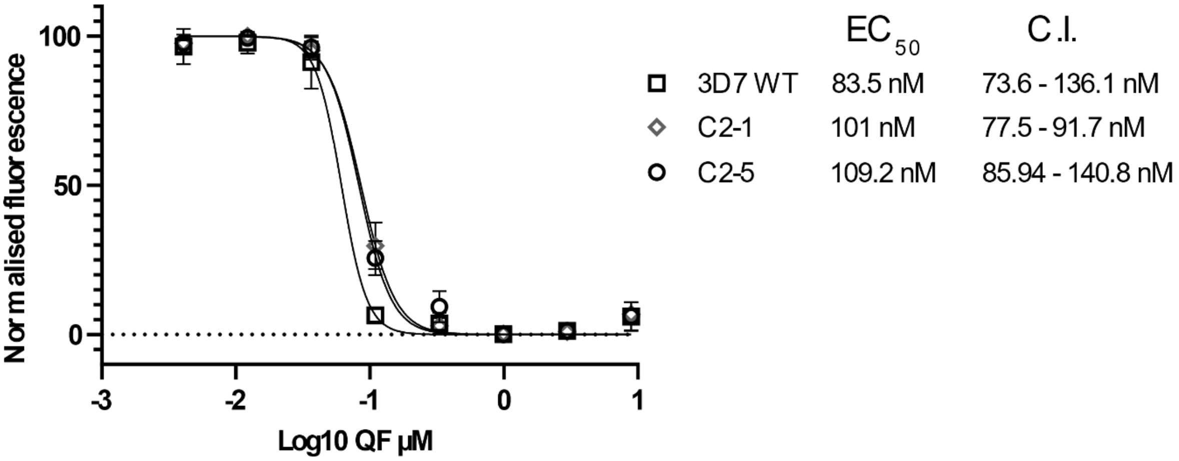

Supplementary Figure 1. Quarfloxin is 'irresistible' in culture.

P. falciparum 3D7 parasites were cultured with increasing concentrations of quarfloxin for nine months, until stable growth was achieved. This was possible only up to ~2X EC_{50}, with further increases causing complete death of the culture. Cultures were then clonally diluted (still in quarfloxin) prior to MSF analysis. Data from two clones, C2-1 and C2-5, are shown here, alongside the untreated 3D7 strain: EC_{50} values are not significantly different.

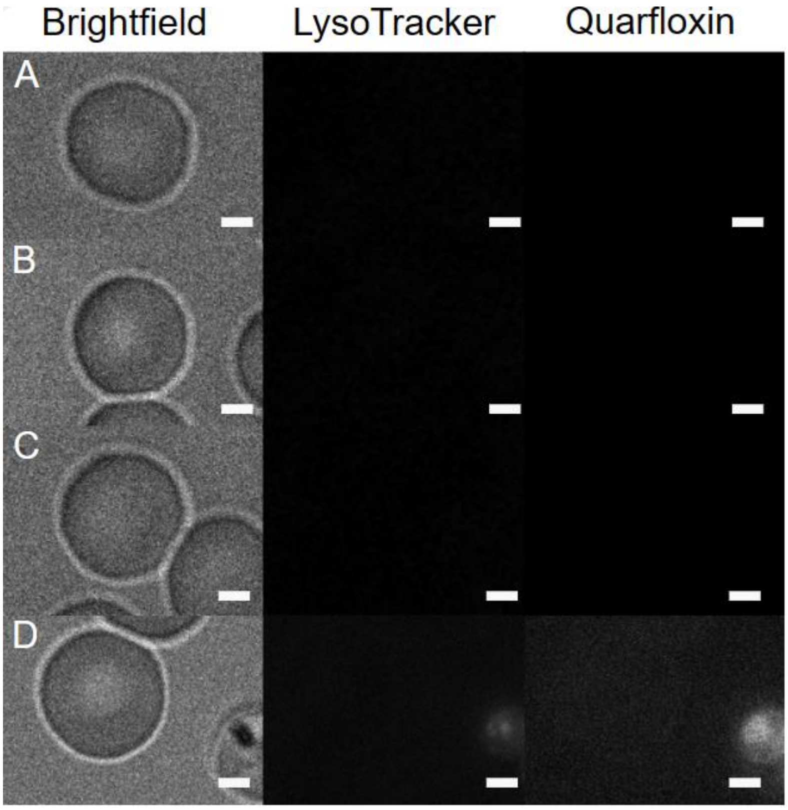

Supplementary Figure 2. Quarfloxin localises less in uninfected RBCs than in parasitised RBCs. *Representative control images of uninfected cells (A-D) and an infected cell (D) stained with quarfloxin and LysoTracker. All images were captured with the same settings and show very little quarfloxin accumulation. Scale bar = 2 µm.*

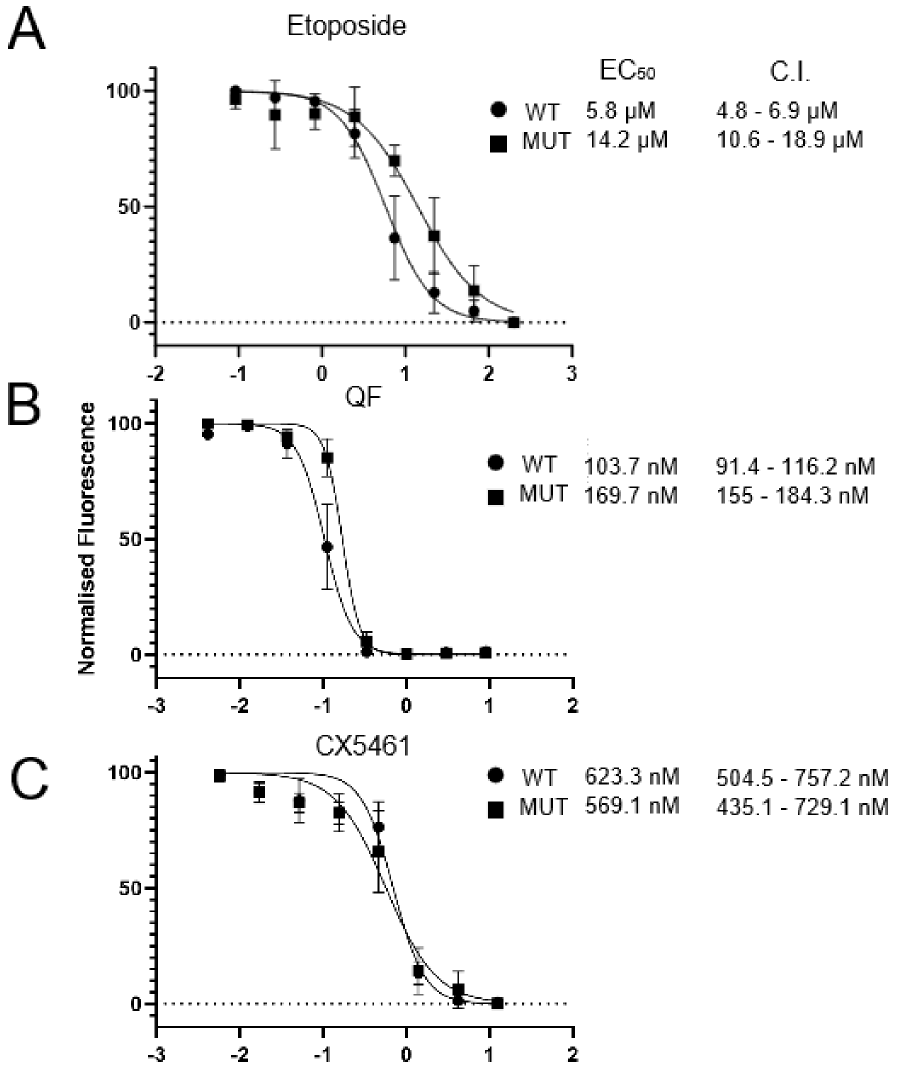

Supplementary Figure 3. Quarfloxin and CX5461 do not primarily act through topoisomerase interaction.

Dose response curve for three drugs on matched parasite lines carrying a Pf RAD54 gene that is either wildtype (WT) or mutated (MUT) at the rG4 locus. The G4MUT line contains increased levels of RAD54 protein, increasing parasite tolerance to etoposide, a topoisomerase targeting compound. A) shows the difference in etoposide sensitivity, ~2.5-fold (statistically significant as indicated by 95% confidence intervals). By contrast, the EC_{50} values on these two lines for quarfloxin (B) differed by ~1.5-fold, and for CX-5461 (C) did not differ.

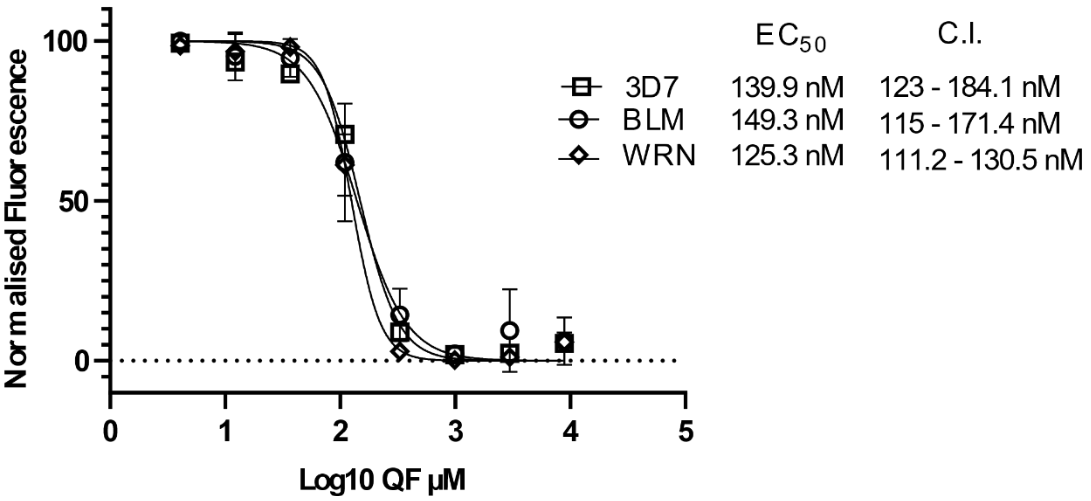

Supplementary Figure 4. Quarfloxin is not significantly more toxic to parasites mutated in the RecQ helicases PfBLM and PfWRN.

Dose responses to quarfloxin were measured in parasite lines mutated in the G4-resolving helicases BLM and WRN. The sensitivities of these lines did not differ significantly from wild-type parasites.

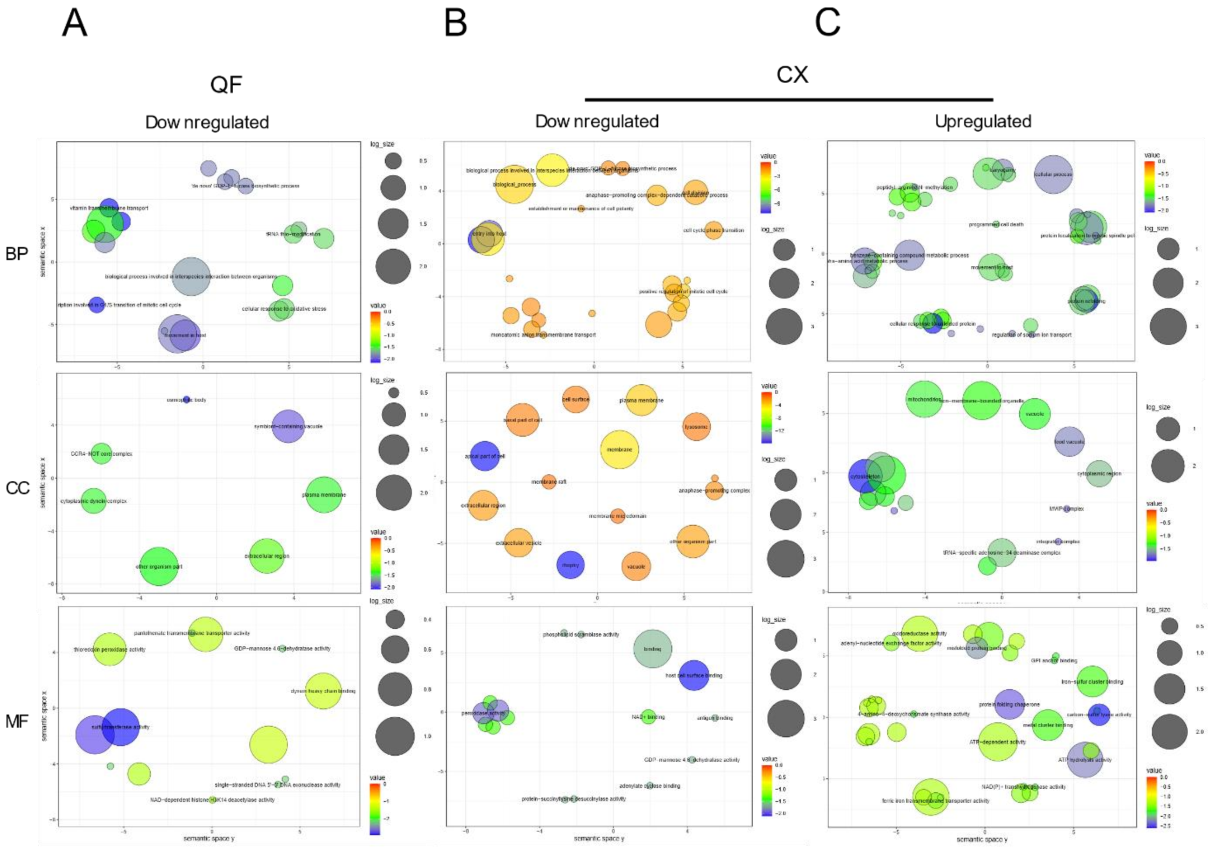

Supplementary Figure 5. GO enrichment analysis of DE genes with ≥2-fold changes. *Biological process (BP), cellular compartment (CC) and molecular function (MF) GO term enrichment was performed for A) quarfloxin downregulated DE genes, B) CX-5461 downregulated and C) CX-5461 upregulated DE genes. Significant terms were plotted in R, P < 0.05.*

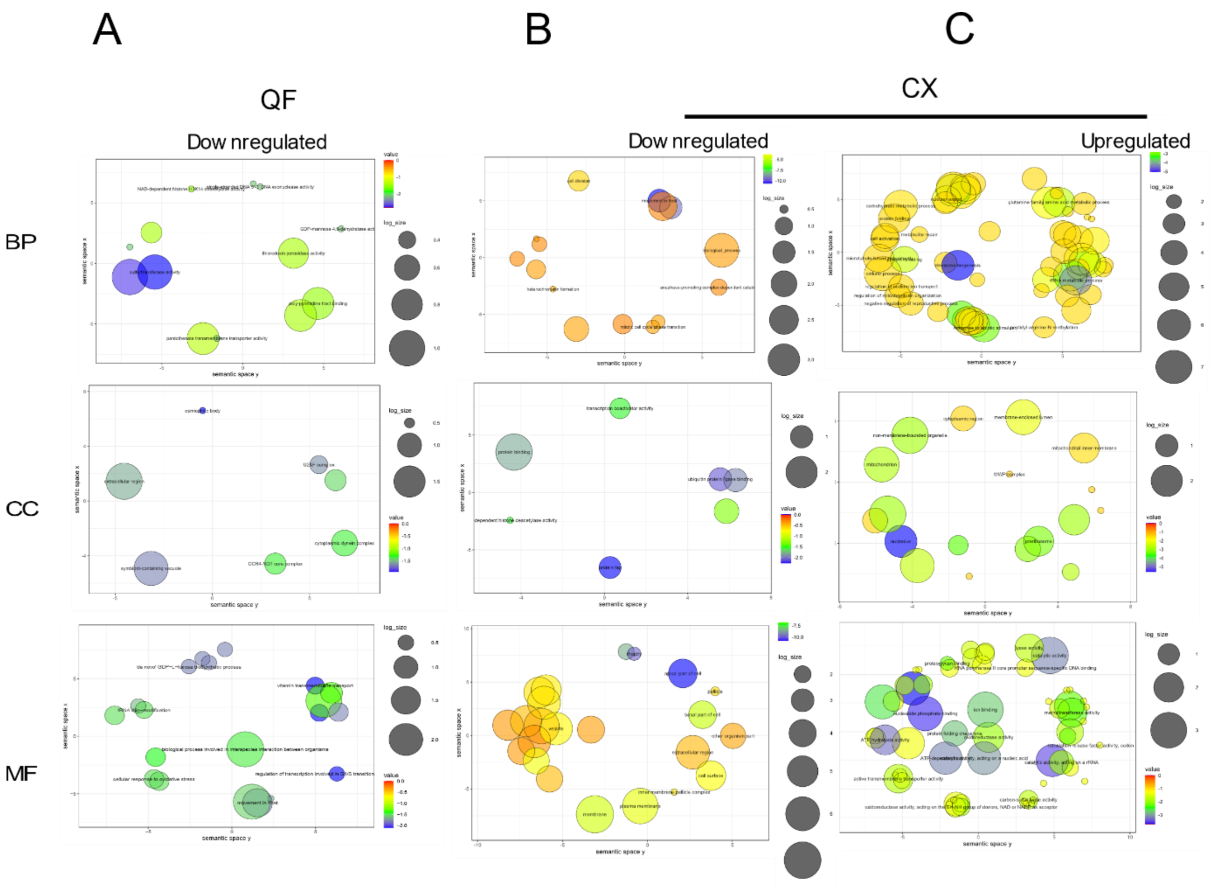

Supplementary Figure 6. GO enrichment analysis of DE genes with ≥1.5x fold changes. *Fold change threshold was reduced to 1.5 and enrichment was repeated as in Supplementary Figure 5. Significant terms were plotted in R, P<0.05.*

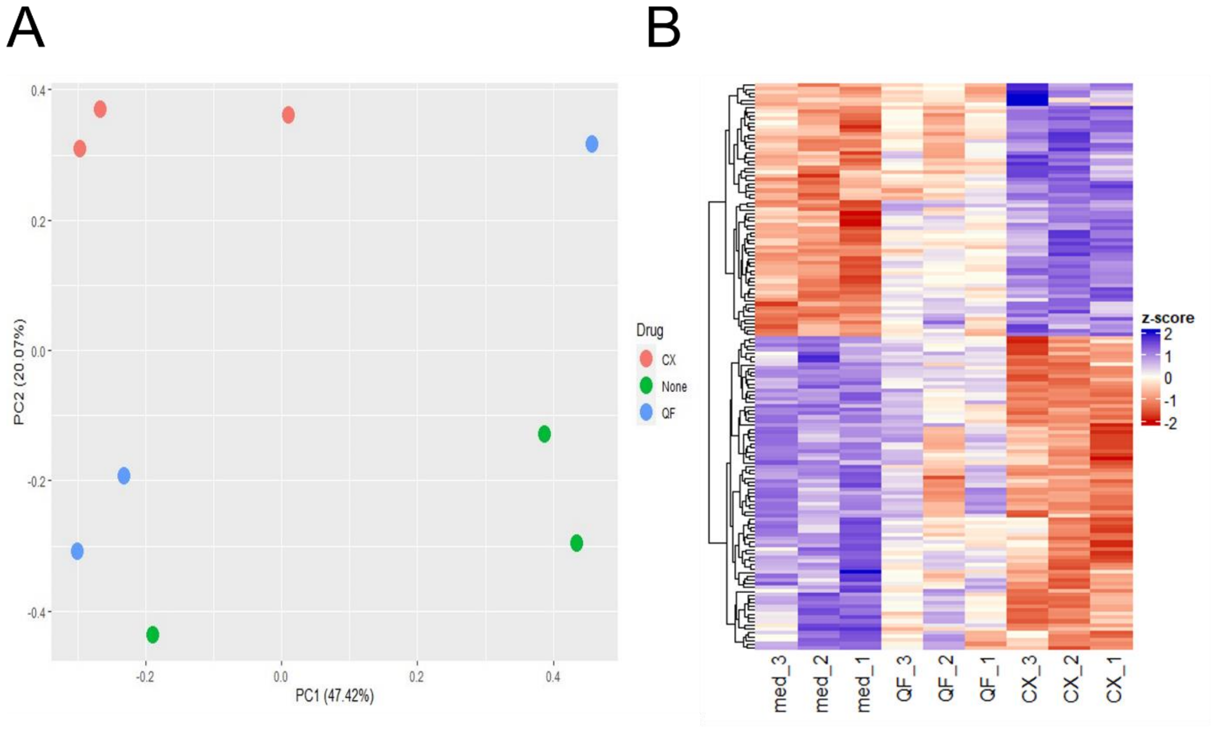

Supplementary Figure 7. Spread of DE data between and within samples.
A) PCA plots show clustering of individual biological repeats (3 repeats per treatment type).
B) Heatmap of top 150 DE genes as determined by FDR. Sample QF2 was removed as outlier.

Legends for Supplementary tables provided as spreadsheet

Supplementary Table 1.
Differentially expressed genes in quarfloxin-treated parasites vs no drug control, adj. P<0.05, log2FC >1.

Supplementary Table 2. *Differentially expressed genes in CX-5461-treated parasites vs no drug control, adj P<0.05, log2FC>1.*

Supplementary Table 3. *Differentially expressed genes that were found to contain G4s in previously published datasets. Datasets used include G4hunter prediction (scoring threshold >1.2) and rG4seq with and without PDS [9, 12].*

Supplementary Table 4. *Oligos used in all experiments and their sequences.*
